# From cytokine storm to adaptive dysregulation: temporal evolution of the inflammatory network and phase-specific intervention strategies in fulminant viral myocarditis

**DOI:** 10.3389/fimmu.2026.1946787

**Published:** 2026-10-19

**Authors:** Yong Liu, Jing Li, Guie Xiao, Changping He

**Affiliations:** 1 Department of General Medicine, the Second Affiliated Hospital of Wannan Medical College, Wuhu, China; 2 School of Medicine, Anhui University of Science and Technology, Huainan, China; 3 Department of Infectious Disease, Shaoyang Central Hospital, Shaoyang, China

**Keywords:** candidate immunoparalysis trajectory, cytokine storm, extracorporeal membrane oxygenation, fulminant myocarditis, innate immunity, precision immunomodulation, viral myocarditis

## Abstract

Fulminant viral myocarditis (FVM) is characterized by acute onset and rapid progression, often deteriorating into cardiogenic shock, malignant arrhythmias, and multi-organ failure within days. Its pathological essence does not lie in the “quantitative accumulation” of focal lymphocytic infiltration seen in ordinary myocarditis, but rather in a “qualitative shift” driven by diffuse myocardial edema compounded by a multi-cytokine cascade. Accumulating evidence indicates that the “inflection point” of myocardial injury is determined not by viral load per se, but by the dysregulated pattern recognition of viral antigens by the innate immune system and the resultant imbalance of the inflammatory network. The organising framework of this review is temporal (hyperacute to acute to reparative immune states). A spatial dimension, contrasting the circulating and the cardiac-resident immune compartments, is developed wherever direct or closely analogous data exist, but it carries far less evidence than the temporal dimension and is treated as secondary to it. Myocarditis that is immune-mediated without a replicating cardiotropic virus, including vaccine-associated and checkpoint-inhibitor disease, is used here as a labelled comparator and not as part of the entity under review. The virus invades cardiomyocytes and vascular endothelium via receptors with non-canonical distribution, subsequently triggering type I interferon responses and necroptosis through endosomal and cytosolic pattern-recognition receptors, and initiating a cascade effect via damage-associated molecular patterns (DAMPs). The driver of cardiomyocyte death does not stay constant across this course: virus-intrinsic cytopathic and ferroptotic death predominates in the first days, myeloid-mediated killing takes over through the remainder of the first week, and cytotoxic lymphocytes become the principal effectors thereafter. The hyperacute phase is characterized by macrophage phenotypic drift, neutrophil extracellular trap (NET) formation, and a “cytokine storm” whose earliest cellular sources in viral myocarditis are myeloid rather than T-cell derived. The acute phase features the “clearance-injury” paradox of cytotoxic T cells and the failure of immune checkpoint regulation, and a subset of patients may subsequently follow what the authors describe as a candidate immunoparalysis trajectory rather than an established third stage of disease - a state provisionally characterized by regulatory T cell (Treg) expansion, paradoxical elevation of interleukin-10 (IL-10), and downregulation of monocyte human leukocyte antigen-DR (HLA-DR). Its reported association with secondary infection and adverse outcome derives largely from sepsis and critical-care cohorts and has not been demonstrated in FVM. Building on this “inflammatory time window,” the authors propose a phase-specific framework intended to generate hypotheses rather than to recommend treatment: in the hyperacute phase, “fire-extinguishing” approaches (complement blockade and cytokine adsorption) may represent a potential alternative to broad-spectrum immunosuppression; in the acute phase, selective blockade of individual pathways according to inflammatory subtype may be worth exploring; and in the reparative phase, measures aimed at restoring regulatory immune capacity may be considered for study. None of these pathways, and none of the biomarker values discussed in the main text, has been prospectively validated in FVM. Finally, this review envisions individualized decision-making guided by multi-omics and dynamic immune monitoring, emphasizing that the prognosis of FVM depends on the capacity for “dynamic phenotyping and real-time switching” of immune status, rather than reliance on any single therapeutic modality.

## 1 Introduction

Myocarditis is defined as inflammatory injury of the myocardium, with etiologies encompassing infectious, immune-mediated, and toxic factors, and a clinical spectrum ranging from silent asymptomatic presentation to fulminant cardiogenic shock (1). Its annual incidence is approximately 4 to 14 cases per 100,000 population, with an overall case-fatality rate between 1% and 7% (2). Global burden of disease data provide a more complete picture: incident cases rose from approximately 780,000 in 1990 to about 1.27 million in 2019, an increase of over 60% (3); although age-standardized incidence and mortality have shown a slow decline, absolute case numbers continue to rise, with notably heightened risk during the COVID-19 pandemic (4). The age distribution of this burden deserves attention—children have a large baseline case load, but mortality has declined significantly over the past three decades (5); individuals aged 30–39 years bear the highest incidence and the heaviest burden of death and disability (6). In other words, this is a disease that predominantly strikes previously healthy individuals.

Within this disease spectrum, fulminant myocarditis (FM) occupies the most severe end. The American Heart Association defines it as an abrupt, severe inflammatory process of the myocardium that can rapidly lead to myocardial necrosis, edema, and cardiogenic shock (7); patients often experience hemodynamic collapse within hours of their first presentation, necessitating inotropic support or even mechanical circulatory support to maintain tissue perfusion (8). To regard FM simply as an “exacerbated form” of ordinary myocarditis would obscure their essential difference: the latter is predominantly characterized by focal lymphocytic infiltration, whereas the former presents with diffuse myocardial edema compounded by a multi-cytokine cascade—a distinction that is not a “quantitative” difference in severity but rather approximates a “qualitative” shift in the organizational pattern of the inflammatory network (9, 10). One frequently cited clinical observation requires the two categories of outcome to be kept separate. In the cohort reported by Ammirati and colleagues, fulminant presentation was associated with substantially higher rates of death or heart transplantation than non-fulminant disease both at 60 days and over seven years of follow-up (11); that study therefore does not support the claim that recovery after shock is more complete. What has been described separately, and far less consistently, is that among patients who survive the acute phase, left ventricular ejection fraction frequently improves during follow-up (13). Recovery of ventricular function in survivors and hard clinical endpoints in the whole cohort are distinct outcomes, and they are kept distinct throughout this review.

This paradox places treatment decisions in a dilemma. If myocardial injury is primarily attributed to the virus, then antiviral therapy and circulatory support should be the mainstays; if it is attributed to the immune response, then potent immunosuppression would seem logical. The clinical reality, however, is that neither approach consistently yields reproducible efficacy, and diagnostic strategies and the timing of drug administration remain largely empirical (7). Although contemporary pathways have emphasized stratified diagnosis—first excluding coronary and valvular etiologies, then establishing the diagnosis through imaging and histology (12)—there remains a persistent lack of actionable criteria for “when to suppress, what to suppress, and to what extent.” A systematic review of fulminant phenotypes has indicated that the true lever for altering outcomes lies in early recognition of decompensation and timely circulatory support (13); this suggests that the long-overlooked critical variable may not be the class of drug, but rather the dimension of time.

This review thus posits a core scientific proposition: the “inflection point” of myocardial injury depends not on viral load, but rather on the dysregulated pattern recognition of viral antigens by the innate immune system. It is the intensity and regulatory quality of the host immune response, rather than the absolute quantity of the pathogen, that determines whether the myocardium proceeds toward controlled inflammation and repair or slides toward uncontrolled tissue destruction (10, 14). Following this logic, neither “anti-inflammatory” nor “immunosuppressive” therapy should be regarded as a fixed strategy applied uniformly throughout the entire course—the same intervention may be life-saving in the hyperacute phase, yet may delay viral clearance and precipitate secondary infections if administered during the reparative phase (15).

### 1.1 Literature search strategy and scope of evidence

To ensure transparency and reproducibility, we searched PubMed/MEDLINE, Embase, Web of Science, and the Cochrane Library from database inception to June 2026, using combinations of the terms “fulminant myocarditis,” “viral myocarditis,” “cytokine storm,” “immunoparalysis,” “innate immunity,” “adaptive immunity,” and “cardiogenic shock,” together with mechanism- and drug-specific terms (e.g., “IL-6,” “TNF-alpha,” “NLRP3,” “tocilizumab,” “vilobelimab,” “hemadsorption,” “low-dose IL-2”), combined with Boolean operators and restricted to English- and Chinese-language publications. Reference lists of key reviews and included articles were hand-searched to identify additional relevant studies. Two authors (Yong Liu and Changping He) independently screened titles and abstracts and then full texts for eligibility; disagreements were resolved by discussion with the corresponding authors. Because direct clinical evidence in fulminant viral myocarditis (FVM) is sparse, we deliberately included mechanistic, preclinical, and cross-disease studies (e.g., immune-checkpoint-inhibitor myocarditis, vaccine-associated myocarditis, giant-cell and eosinophilic myocarditis, COVID-19, sepsis, ischemic heart disease, and experimental cardiac fibrosis models) where these provided the only available mechanistic or therapeutic rationale; such evidence is explicitly identified as extrapolated rather than FVM-specific throughout the text, in **Table 1**, and in the discussion of each intervention in Section 5. This review does not follow a formal PRISMA-based systematic methodology and should be regarded as a structured narrative synthesis; where feasible, we graded the supporting evidence informally as randomized controlled trial, prospective/retrospective cohort or registry, case series/case report, or preclinical/mechanistic, and this hierarchy is made explicit in the “Level of evidence” column of **Table 1** and in the text accompanying each recommendation.

**Table 1:** Evidence summary of immune-targeted agents in fulminant/acute myocarditis.

Agent	Target	Suggested immune phenotype	Level of evidence	Key caution	Ref.
Methylprednisolone (pulse)	broad (NF-κB)	virus-neg, high IL-6, unstable	RCT (chronic, virus-neg): TIMIC [different regimen/population from acute FVM; extrapolated]	harmful if virus+/paralysis	(136, 137)
Tocilizumab	IL-6R	IL-6-dominant	case reports/RCT in COVID CRS	infection; masks CRP; extrapolated from ICI-myocarditis/COVID-19, not FVM-specific	(132, 134, 163)
Etanercept	TNF-α	TNF-dominant, no shock	case reports (salvage)	TNF has host-defense role; negative RCT (RENEWAL) in chronic HF (203)	(135)
IVIG	polyclonal	acute/fulminant (esp. pediatric)	retrospective/neutral RCTs	no mortality benefit (adult)	(166, 167, 201)
Anakinra	IL-1R	high-inflammatory/NLRP3	case series; RCT (ARAMIS) neutral	benefit unproven in all-comers	(139, 153, 154)
Vilobelimab	C5a	hyperacute, neutrophil-driven	phase 3 in COVID ARDS	no direct myocarditis RCT	(126)
CytoSorb (hemadsorption)	cytokines (IL-6)	hyperacute, high cytokine load, ECMO	case series/retrospective	hard endpoints unproven	(129)
Abatacept + ruxolitinib	CD86 (CTLA-4) + JAK	ICI-associated, refractory	case series (personalized)	immunosuppression; infection	(195, 196)
Rituximab/plasma exchange	B cells/drug removal	ICI-associated, refractory	case series	delayed onset (rituximab)	(164, 165)
Low-dose IL-2	Treg (CD25)	repair phase/immunoparalysis	early-phase RCTs (IHD/ACS)	capillary leak at high dose	(143)
FAP CAR-T (experimental)	fibroblast activation protein	repair/fibrotic (experimental)	preclinical only	not yet in humans	(174, 175)
MSC-exosome (miR-146a)	IRAK1/NF-κB	repair phase (experimental)	preclinical only	not yet in humans	(145)
MCC950	NLRP3	pyroptosis-driven (experimental)	preclinical only	not yet in humans	(69)

Level of evidence is deliberately shown to expose the observational nature of most data.

VA-ECMO, venoarterial extracorporeal membrane oxygenation; ICI, immune checkpoint inhibitor; CRS, cytokine release syndrome; RCT, randomized controlled trial; FAP, fibroblast activation protein; IHD, ischemic heart disease; ACS, acute coronary syndrome.

To keep this distinction visible rather than buried, we apply a consistent three-tier convention throughout the remainder of the manuscript. Evidence obtained directly in viral or fulminant myocarditis cohorts is presented without qualification. Evidence from other cardiovascular conditions — chronic virus-negative inflammatory cardiomyopathy, chronic heart failure, ischemic heart disease, or experimental cardiac fibrosis — is identified as indirect cardiovascular evidence at the point of citation. Evidence from non-cardiac inflammatory states, including COVID-19, sepsis, oncologic cytokine-release syndrome, and systemic autoimmune disease, is identified as cross-disease extrapolation and is framed as mechanistic reference rather than as grounds for a therapeutic recommendation in FVM. Where a proposed intervention rests wholly on the third tier, we say so explicitly in the relevant subsection of Section 5 and in the corresponding row of **Table 1**. We retain fulminant viral myocarditis as the stated scope of this review rather than broadening the title to fulminant myocarditis in general, because the virus-positive versus virus-negative distinction is precisely what governs the safety of immunosuppression and is therefore central to the argument developed here; the cost of that choice is a thinner direct evidence base, which the convention above is intended to keep transparent.

Two further conventions govern how evidence from outside viral myocarditis enters the argument. The first concerns where on the disease timeline such evidence is allowed to land. Viral myocarditis follows a sequence: viral entry and direct myocardial injury, exposure of cardiac antigen, innate and then adaptive immune activation, and, in a minority of patients, persistent immune-mediated inflammation after the virus is no longer detectable. Autoimmune myocarditis and immune-checkpoint-inhibitor myocarditis model the far end of that sequence, since in both the myocardium is virus-free and the pathology is driven by antigen-specific lymphocytes. Evidence from those conditions is therefore applied in this review chiefly to the acute and reparative intervals and to the transition toward chronic inflammatory cardiomyopathy. Where it is invoked for hyperacute events, the text says so and marks the inference as weak, because in the first days the myocardium is still virus-positive and the dominant effectors are myeloid rather than clonally expanded T cells. The second convention concerns layout. In the subsections where direct and indirect evidence are most easily conflated - Sections 2.5, 3.3, 4.1 and 4.3 - the argument is set out in three explicitly labelled layers: evidence established in viral or fulminant myocarditis; cross-disease evidence and mechanistic extrapolation; and the synthesis or hypothesis that the two layers jointly support. A reader who wants only the empirical base can stop at the first layer of each of those subsections.

## 2 Initiating events: viral invasion and the “alert storm” of DAMPs/PAMPs

### 2.1 Non-canonical distribution of viral receptors: endothelium as an inflammatory “amplifier”

The heart is not an isolated target organ for viruses. Taking coxsackievirus B3 (CVB3) as an example, it invades cardiomyocytes via the coxsackievirus-adenovirus receptor (CAR) and decay-accelerating factor (DAF) as co-receptors. CAR, localized at the intercalated discs of cardiomyocytes, is critical for viral internalization (16). Of note, CAR is highly expressed in fetal and neonatal hearts and is markedly downregulated after birth, which partly explains the high susceptibility of young individuals to CVB3 infection (17). In the myocardium of patients with dilated cardiomyopathy and myocarditis, CAR expression is frequently upregulated and correlates with viral load and adverse outcomes, suggesting that both the virus and the receptor jointly contribute to disease progression (18, 19). CAR gene polymorphisms have also been considered as potential host factors for individual susceptibility (20).

For severe acute respiratory syndrome coronavirus 2 (SARS-CoV-2), angiotensin-converting enzyme 2 (ACE2) serves as its primary receptor. Both cardiomyocytes and vascular endothelium highly express ACE2. The virus enters cells via spike protein binding to ACE2, which subsequently downregulates ACE2 and promotes angiotensin II accumulation, driving inflammation, oxidative stress, and adverse myocardial remodeling through the angiotensin II type 1 receptor (AT1R) (21, 22). Given that endothelial cells highly express the above receptors, the vascular bed serves as an inflammatory “amplifier.” Parvovirus B19 (B19V) even specifically targets endothelial cells of intramyocardial arterioles and post-capillary venules, rather than cardiomyocytes, and upregulates proinflammatory cytokines and induces endothelial apoptosis through its nonstructural protein NS1, leading to endothelial dysfunction and persistent myocardial inflammation (23, 24). Over the past decade, B19V and human herpesvirus 6 (HHV-6) have replaced enteroviruses and adenoviruses as the most commonly detected pathogens in endomyocardial biopsy specimens. Their role as “bystanders” or “causative agents” needs to be distinguished by considering viral load, genotype, and transcriptional activity (25, 26).

### 2.2 Overactivation of intracellular pattern-recognition receptors: cardiomyocytes as “non-professional immune cells”

Once viral nucleic acids enter the cell, they are recognized by endosomal Toll-like receptors (TLR3, TLR7/8) and cytosolic RIG-I-like receptors (RIG-I, MDA5). Both pathways converge at the mitochondrial antiviral signaling protein (MAVS), which activates TBK1, interferon regulatory factors (IRFs), and nuclear factor-κB (NF-κB), driving the expression of type I interferons and proinflammatory cytokines (27). In CVB3 infection models, TLR3-deficient mice exhibit higher mortality and more severe myocarditis, whereas transgenic overexpression of TLR3 confers protection even in the context of defective type I interferon signaling, highlighting the central role of nucleic acid sensing pathways in early antiviral defense (27). Cardiomyocyte-specific knockout of the type I interferon receptor delays viral clearance and accelerates myocardial pathology, further demonstrating that interferon signaling is indispensable for restricting viral spread within the myocardium (28). The key point here is that cardiomyocytes themselves are “non-professional immune cells.” Upon activation, cardiomyocytes can release large amounts of type I interferons (IFN-α/β), which constitutes the first domino in the cascade effect (29). Taking the encephalomyocarditis virus model as an example, MDA5-mediated interferon responses are crucial for limiting viral replication and protecting the myocardium, and the strength of this signaling directly determines the trajectory of myocardial injury (30). In SARS-CoV-2 infection, although RIG-I can trigger antiviral defense, the virus can evade this recognition through multiple strategies, leading to a temporal mismatch in the innate immune response (31, 32).

### 2.3 Necroptosis and DAMP release: escalation from local to systemic inflammation

When cardiomyocytes undergo programmed necrosis such as necroptosis, damage-associated molecular patterns (DAMPs), including high-mobility group box 1 (HMGB1), heat shock proteins, and mitochondrial DNA (mtDNA), are released into the extracellular space (33). Among these, HMGB1 amplifies cytokine release via the TLR4 and NF-κB pathways (33); while mtDNA that escapes into the cytosol or extracellular space activates the cyclic GMP-AMP synthase (cGAS)–stimulator of interferon genes (STING) pathway, escalating local inflammation to systemic inflammation (34). In a CVB3 myocarditis model, mtDNA released from infected cardiomyocytes drives inflammatory responses in cardiac macrophages through a STING-dependent mechanism, and STING deficiency significantly attenuates this response (34). This axis—”mitochondrial damage → mtDNA escape → cGAS-STING → NF-κB”—has also been observed in diabetic cardiomyopathy, immune checkpoint inhibitor-associated myocarditis, and other conditions, suggesting it represents a common amplification mechanism for sterile inflammation in the myocardium (35, 36).

### 2.4 The spectrum of viruses that cause myocarditis: tropism, detection and attribution

The viruses that cause myocarditis are most usefully grouped by the cell they occupy once they reach the heart, because that choice of host cell determines how injury is produced. Enteroviruses, and coxsackievirus B3 in particular, remain the prototype. They enter cardiomyocytes through CAR with DAF as co-receptor, replicate within them, and generate the classical sequence of direct cytopathic injury followed by mononuclear infiltration; CAR expression is high in the fetal and neonatal heart, falls after birth, and is re-expressed in myocarditic and dilated myocardium in proportion to viral load and adverse outcome (16–19). Adenovirus uses the same receptor, and together with the enteroviruses it dominated biopsy series through the 1990s (25). What is recovered from endomyocardial biopsy today is different. Parvovirus B19 and human herpesvirus 6 head most contemporary European series, and B19V is not a cardiomyocyte virus at all: it occupies endothelial cells of intramyocardial arterioles and post-capillary venules, where its NS1 protein induces endothelial apoptosis and cytokine release, so that myocardial damage arises through endothelial dysfunction and secondary inflammation rather than through lysis of infected myocytes (23–26, 49). Both agents can persist in myocardium transcriptionally silent, which is why a positive genome result cannot by itself be called aetiological; viral load, genotype and evidence of active transcription have to be weighed before the bystander interpretation is discarded (25, 26). Influenza A occupies a third position. It infects cardiomyocytes and the conduction system directly, and its fulminant presentations include transient ventricular wall thickening with tamponade as well as biventricular thrombosis on a background of ventricular dysfunction and hypercoagulability (43–45). SARS-CoV-2 sits at the opposite end of the spectrum from CVB3. Autopsy series detect viral genome in myocardium only rarely, macrophage and T-cell infiltrates are also found in non-infectious deaths, and myocardial injury in hospitalised patients is attributable mainly to hypoxia, pre-existing cardiac disease and systemic inflammation rather than to invasion of cardiomyocytes (37, 38); the reported prevalence of 2.3% to 5.0% therefore aggregates direct, immune-mediated and mimicry mechanisms and should not be read as a rate of virus-positive myocarditis (39). Chagas cardiomyopathy is retained only as a differential diagnosis in the relevant epidemiological setting: Trypanosoma cruzi is a parasite, its cardiac disease is chronic, and the mechanisms invoked for it - parasite persistence, autoimmunity, microvascular disturbance and autonomic damage, together with endothelin-1-dependent calcium/calcineurin/NFAT activation and elevated macrophage migration inhibitory factor - are cited here for contrast rather than as part of the fulminant viral spectrum (46–48).

Myocarditis after mRNA vaccination is deliberately excluded from this spectrum. No replicating cardiotropic virus is present in the myocardium; the epidemiology, concentrated in young males within days of a second dose, and the mechanisms proposed for it, molecular mimicry directed at the spike protein and unmasking of pre-existing immune dysregulation, place it with the immune-mediated rather than the viral myocarditides (40–42). For that reason, vaccine-associated disease is not reviewed here in detail. Where vaccine-associated or checkpoint-inhibitor myocarditis appears later in the text, it appears as an explicitly labelled cross-disease comparator and nowhere as evidence about viral myocarditis itself.

### 2.5 Cardiomyocyte death in FVM: which driver dominates, and when

The pathways below are discussed because each acts as a trigger or an amplifier of the DAMP-driven cascade introduced in Section 2.3, not as an inventory of ways a cardiomyocyte can die. Listing them in parallel leaves the question a clinician actually asks unanswered: at a given point in the illness, is the myocardium being destroyed by the virus or by the response to it? That question is addressed at the end of this subsection, after the individual pathways have been set out, and the answer is organised in the three evidence layers described in Section 1.1.

Cardiomyocytes are not limited to only necrosis and apoptosis as their death pathways. Ferroptosis, an iron-dependent form of lipid peroxidation-driven cell death, has been demonstrated to participate in viral myocarditis: coxsackievirus B3 can cleave the selective autophagy receptor p62, inactivate the KEAP1/NRF2/GPX4 axis, thereby driving ferroptosis and promoting its own replication, whereas a selenium-rich diet attenuates this injury by supplementing the GPX4 substrate (50). GPX4 serves as the core gatekeeper of ferroptosis; its deficiency exacerbates various forms of cardiac injury (51), and the regulatory network of ferroptosis in cardiovascular diseases is increasingly well delineated (52). Intriguingly, STING can target GPX4 for autophagic degradation, directly coupling innate immune signaling to ferroptosis (53). Autophagy, in turn, acts as a double-edged sword. As a positive-strand RNA virus, coxsackievirus B3 hijacks the autophagic machinery to support its replication: inhibition of autophagosome formation significantly reduces viral titers (54), and autophagosome-like vesicles can be observed in the myocardium *in vivo* (55). Its non-structural proteins, together with glycolytic reprogramming, sustain autophagic flux (56, 57); however, the virus concurrently blocks autophagosome-lysosome fusion by downregulating syntaxin 17, resulting in autophagosome accumulation (58). Some studies have questioned whether autophagosomes serve as replication platforms, suggesting the existence of autophagosome-independent replication mechanisms (59). These seemingly contradictory observations precisely indicate that the role of autophagy is highly dependent on the viral strain and disease stage, making generalization difficult, and thus interventions targeting autophagy still require caution (60).

Pyroptosis directly couples cell death with inflammatory amplification. The NLRP3 inflammasome, via caspase-1-mediated cleavage of GSDMD, has been confirmed as an important form of cardiomyocyte death in animal models of myocarditis (61); the multifaceted roles of the inflammasome in viral myocarditis have also been systematically reviewed (62). Upstream triggering mechanisms are being progressively dissected: mitochondrial calpain-1 can activate NLRP3 (63), cathepsin B release exacerbates coxsackievirus B3 myocarditis (64), and canonical NLRP3 inflammasome activation is indispensable for CVB3-induced myocardial injury (65). Human myocardial samples have also confirmed the activation of inflammasome signaling (66). At a broader level, pyroptosis, apoptosis, and necroptosis can be concurrently orchestrated through the ZBP1/RIG-I-triggered “PANoptosome,” with GSDMD pore formation facilitating mtDNA egress and feeding back into the cGAS-STING pathway, thereby constituting an amplification loop (67). This network also provides therapeutic entry points: the small molecule MG53 attenuates pyroptosis by inhibiting the NF-κB pathway (68), and selective NLRP3 inhibitors have shown potential to mitigate injury in ischemic myocardial models (69).

Established evidence in viral myocarditis. Two findings anchor the temporal question. First, direct cytopathic death is virus-driven and early, and the virus actively recruits the host death machinery to its own ends: CVB3 cleaves p62, inactivates the KEAP1/NRF2/GPX4 axis and drives ferroptosis in a way that promotes rather than limits its replication, and it sustains autophagic flux while blocking autophagosome-lysosome fusion (50, 54–58). Canonical NLRP3 activation is required for CVB3-induced myocardial injury, and inflammasome signalling is demonstrable in human myocardial tissue (61–66). Second, the virological and the histological peaks of the disease are separated. In murine CVB3 infection, myocardial viral titre peaks within the first days and then declines, whereas mononuclear infiltration and the maximum of histological injury follow in the second week; loss of TLR3 or of cardiomyocyte type I interferon signalling shifts both curves and worsens outcome, which is only interpretable if the two peaks are mechanistically distinct (27, 28). Effectors capable of killing uninfected myocytes are documented in that later window: perforin release onto cardiomyocyte membranes was shown directly by immunoelectron microscopy in myocarditis (91), NK cells limit CVB3 and cytomegalovirus myocarditis, and their suppression by myeloid-derived suppressor cells raises viral load and worsens injury (92, 93).

Cross-disease evidence and mechanistic extrapolation. What has never been done in human FVM is the quantitative attribution of cardiomyocyte death to a named effector at a named time point. Evidence from checkpoint-inhibitor myocarditis supplies the clearest picture of T-cell-mediated myocyte killing: clonally expanded, granzyme-B-high CD8+ T cells dominate the infiltrate, granzyme B cleaves myocardial structural proteins, and depletion of CD8+ but not CD4+ cells rescues survival in Pdcd1-/-Ctla4+/- animals (99–101). Experimental autoimmune myocarditis adds that anti-myosin CD4+ responses alone suffice to produce substantial inflammation with no infectious trigger (104). Both models are, by construction, virus-free. Their informative value for FVM is therefore confined to the interval in which viral replication has already waned, and applying them to the first 72 hours would invert the actual sequence of events.

Synthesis. Combining the two layers, we propose an explicit temporal partition of cardiomyocyte death in FVM, drawn in the new panel B of **Figure 1**. Through roughly the first 72 hours the dominant driver is virus-intrinsic: cytopathic replication, replication-serving ferroptosis, and pyroptosis of infected myocytes, with the DAMPs so released recruiting the myeloid compartment. Across the remainder of the first week the balance moves to innate immune killing - histone cytotoxicity from NETs, macrophage effector products, complement activation - which is the first mechanism in the sequence capable of damaging myocytes that were never infected. From approximately day 7 to day 14, cytotoxic CD8+ T cells and NK cells become the principal executioners, and the clearance-injury paradox of Section 4.1 belongs here rather than at presentation. Beyond two weeks, cell death falls to a low-grade pyroptotic and autophagic level that feeds fibrotic remodelling instead of acute pump failure. The clinical corollary is the reason for stating the partition at all: antiviral and cytoprotective strategies can only act within the first window, whereas immunosuppression can only act within the second and third, so a therapy given at the wrong point in this sequence is not merely less effective but acting on a process that is no longer dominant. We say plainly that the partition is inferred. It rests on murine CVB3 time-course data and on the separation of virological and histological peaks in human biopsy material, not on serial sampling of human FVM, and the study that would test it - paired quantification of viral load and of infiltrate composition on sequential biopsies, or on tissue obtained at the time of mechanical support - is among the most tractable questions this review raises.

**Figure 1:**
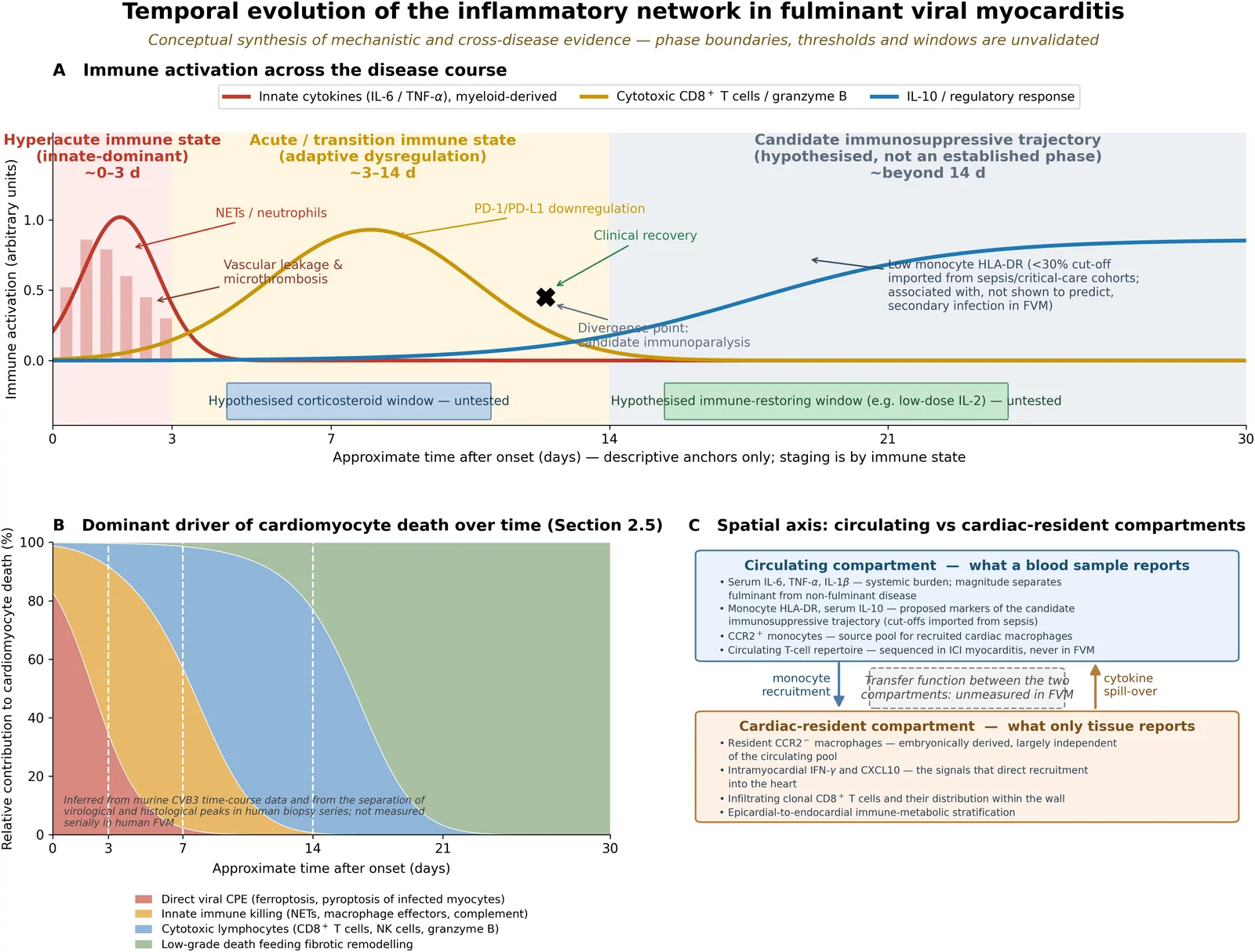
Temporal evolution of the inflammatory network in fulminant viral myocarditis, with the death-driver and compartment axes shown explicitly. **(A)** Immune activation against time. Calendar intervals are approximate descriptive anchors rather than natural-history boundaries; the states are defined by immune phenotype. The hyperacute state (approximately 0–3 d) is dominated by a myeloid-derived cytokine surge (IL-6/TNF-alpha) and NET release with vascular leakage and microthrombosis; the acute state (approximately 3–14 d) shows the peak of cytotoxic CD8+ T cells with checkpoint dysregulation; thereafter the course diverges toward clinical recovery or toward a candidate immunosuppressive trajectory marked by rising IL-10 and low monocyte HLA-DR. The <30% HLA-DR cut-off is imported from sepsis and critical-care cohorts and is associated with, rather than demonstrated to predict, secondary infection in FVM. **(B)** The shifting dominance of the mechanisms that kill cardiomyocytes: direct virus-induced cytopathic and ferroptotic death early, innate myeloid-mediated killing through the remainder of the first week, cytotoxic lymphocyte-mediated killing from approximately day 7, and low-grade death feeding fibrosis thereafter (see Section 2.5). The crossover is inferred from murine CVB3 time-course data and from the separation of virological and histological peaks in human biopsy material; it has not been measured by serial sampling in human FVM. **(C)** Summary of the spatial axis: what is measurable in the circulating compartment, what is measurable in the cardiac-resident compartment, and the gap between them, which remains the least developed dimension of this review (Section 3.1). The bottom bars in panel A indicate hypothesised, untested benefit windows for corticosteroids and for immune-restoring agents. All boundaries, thresholds and windows shown are a conceptual, hypothesis-generating synthesis rather than clinically validated cut-offs (see Section 7).

## 3 The innate “explosion”: cytokine dynamics of the hyperacute phase (approximately the first 72 hours)

### 3.1 Macrophage phenotypic drift and the spatial axis: circulating versus cardiac-resident compartments

In the hyperacute phase, the conventional M1/M2 polarization dichotomy is insufficient to characterize the complex states of macrophages in the myocardial interstitium. Single-cell sequencing has revealed that cardiac macrophages can be divided into at least two major lineages—tissue-resident (CCR2^-^) and monocyte-derived recruited (CCR2^+^)—which exert distinct effects on monocyte recruitment and fate determination following injury (70). Selective depletion of resident CCR2^-^ or CCR2^+^ macrophages produces divergent effects on left ventricular function, myocardial remodeling, and monocyte recruitment (70). In pressure overload and inflammatory cardiomyopathy models, single-cell atlas studies have further identified multiple novel subpopulations, including interferon-inducible, proliferative, and lipid-associated subsets, whose early infiltration into the myocardial interstitium remodels the local inflammatory microenvironment (71, 72).

This phenotypic plasticity of macrophages directly influences disease trajectory. In CVB3 infection, macrophages can be potently activated without viral replication, and their CAR expression status modulates the direction and intensity of inflammatory responses (73, 74). Evidence from vaccine-associated myocarditis, cited here purely as a comparator and not as viral myocarditis, indicates that the monocyte compartment can be metabolically reprogrammed during myocardial inflammation: circulating classical monocytes show altered JUN/FOS activity and remodeling of metabolic-inflammatory gene programs, suggesting that metabolic reprogramming of the monocyte–macrophage axis may participate in the initiation and amplification of myocardial inflammation (75).

The spatial axis needs to be stated in full, together with an honest account of where it runs out of evidence. Its anatomical basis is not in doubt. The myocardium contains a resident macrophage population (CCR2-) that is largely independent of the circulating monocyte pool and a recruited population (CCR2+) that is derived from it, and selective depletion of one or the other produces divergent effects on ventricular function, remodelling and further monocyte recruitment (70). Single-cell atlases of pressure overload and inflammatory cardiomyopathy resolve this further into interferon-inducible, proliferative and lipid-associated subsets whose arrival reshapes the local microenvironment (71, 72). A cell that has sat in the myocardium since embryonic life and a cell that arrived from the blood three days ago are therefore not interchangeable, and a peripheral blood sample reports directly on only one of them.

The same division applies to the mediators. Serum IL-6, TNF-alpha and IL-1beta describe a systemic state, whereas the transcripts that dominate within myocardial tissue are interferon-gamma and CXCL10, which are what actually direct immune cells into the heart (22, 84). The two compartments need not move together, and nothing in the current literature establishes the transfer function between them in FVM: no study has reported paired serum and myocardial cytokine measurements from the same patient at the same time point. Spatially resolved transcriptomics has begun to show that the tissue compartment is itself not uniform. Molecular perturbation in human myocarditis extends well beyond the leukocyte infiltrate, with cardiomyocytes themselves expressing immune-response genes previously considered the preserve of professional antigen-presenting cells, and in giant cell myocarditis the immune-metabolic state is stratified along the epicardial-to-endocardial axis (191–194). A biopsy is thus a sample not only of one organ but of one depth within it, which is one more reason that a single negative fragment cannot exclude the process (Section 6.4).

Where the axis remains weakest is the relationship between the two compartments at the level of individual clones. Overlap between myocardium-infiltrating and circulating CD8+ clones has been characterised in checkpoint-inhibitor myocarditis (99, 100) and, to our knowledge, nowhere in viral myocarditis; whether the blood reports faithfully on the myocardial repertoire in FVM is therefore an open question rather than an assumption we are entitled to make. We have accordingly removed the spatiotemporal claim from the title, since the temporal dimension carries the great majority of the evidence presented here, and we retain the spatial dimension as a secondary, explicitly hypothesis-generating axis that is developed at three points in the review: the resident-versus-recruited macrophage division here, the blood-versus-tissue clonal question in Section 4.1, and the intramyocardial regional structure in Section 6.4. The measurements that would convert it into a primary axis are specifiable: paired serum and tissue cytokine panels, paired peripheral and myocardial T-cell repertoire sequencing, and spatial transcriptomics on biopsy material obtained during the fulminant phase rather than at explant or autopsy.

### 3.2 The “double-edged effect” of neutrophil extracellular traps

Neutrophil extracellular traps (NETs) are web-like structures composed of decondensed chromatin and granule proteins released by activated neutrophils (76). On one hand, NETs can capture pathogens; on the other hand, they cause direct cytotoxic damage to cardiomyocytes and endothelium through histone-mediated mechanisms (77). More critically, NETs provide an anchoring platform for platelets and coagulation factors, can activate coagulation factor XII, and initiate the intrinsic coagulation pathway, thereby inducing microvascular thrombosis and endothelial injury, which constitute the molecular basis for coagulation abnormalities and the “vascular leakage–microthrombosis” phenotype (78, 79).

In ischemic and inflammatory cardiac injury, the “double-edged” nature of NETs is particularly evident: NET burden in coronary thrombus and myocardial infarction patients correlates with infarct size and poor ST-segment resolution, and NET clearance can attenuate myocardial injury (80, 81); meanwhile, NETs can “license” macrophages to produce cytokines, further amplifying inflammation (82). In the context of fulminant myocarditis, NETs serve both as a component of antiviral defense and as a driver of microvascular thrombosis and endothelial injury, with the net effect depending on the timing and intensity of their release (83).

### 3.3 The cytokine storm of viral myocarditis: what has been measured, and what is inferred

Established evidence in viral myocarditis. The cells that generate the early cytokine burden in viral myocarditis are myeloid, not lymphoid, and the evidence for this is direct. Macrophages are potently activated by CVB3 without supporting productive replication, and their CAR expression status sets the direction and the intensity of that response (73, 74). Mitochondrial DNA released from infected cardiomyocytes activates cardiac macrophages through STING, an effect abolished by STING deficiency (34). Neutrophils arrive in the same window and release NETs whose histone component is directly cytotoxic to cardiomyocytes and endothelium and which provide the platform for factor XII activation and microthrombosis (76–79). Inflammasome signalling has been demonstrated in human myocardial samples, and canonical NLRP3 activation is indispensable for CVB3-induced injury (61–66), which places IL-1beta among the cytokines produced *in situ* by the myeloid compartment rather than imported from the circulation. Two levels must then be kept apart when these measurements are read. Systemically, serum IL-6, TNF-alpha and IL-1beta rise, and the magnitude of that rise is one of the features that distinguishes fulminant from non-fulminant disease (9, 10). Within the myocardium, the dominant signals are interferon-gamma and CXCL10, which govern the directed recruitment of the cells that subsequently generate the systemic signal (22, 84). A serum cytokine panel is therefore a downstream echo of an intramyocardial process, and the two levels are not interchangeable.

Cross-disease evidence and mechanistic extrapolation. How an IL-6 response becomes self-sustaining, and what it does to the heart once it has, is best documented outside viral myocarditis. IL-6 signals through membrane-bound and soluble receptors, gp130 and the downstream JAK/STAT3, RAS/RAF and PI3K/AKT pathways, producing endothelial activation, hypotension, complement and coagulation activation and depression of myocardial contractility (88). In SARS-CoV-2 infection the nucleocapsid and spike proteins induce monocyte-macrophage IL-6 secretion, anti-nucleocapsid antibodies amplify it, and occupancy of ACE2 downregulates IRAK-M and so removes one of the brakes on cytokine production (85–87). Cytokine release syndrome after CAR-T therapy is the human setting in which IL-6-driven cardiotoxicity has been characterised most precisely and in which IL-6 blockade was validated (89). It is cited here for one narrow purpose only: it establishes that a systemic IL-6 burden above a certain level is sufficient to depress myocardial function reversibly in humans. It is not a mechanistic template for the first days of viral myocarditis, because CRS is initiated by engineered T cells whereas the early FVM infiltrate is dominated by macrophages and neutrophils, and that difference in cellular source limits how far the analogy can be taken.

Synthesis. Read together, the two layers support a compartment-aware rather than a unitary model of the cytokine storm in FVM: an intramyocardial, myeloid-generated, interferon-and-chemokine-dominated process that recruits further effectors, and a systemic spill-over whose IL-6 component is capable of independent haemodynamic harm. The stratification of patients into IL-6-dominant and TNF-dominant subtypes, proposed by analogy with cluster analyses of severe COVID-19 and other cytokine-storm syndromes, follows from this model but has not been demonstrated in FVM and is labelled throughout as a conceptual extrapolation (90). What is missing is not another mechanism but a measurement: serial, compartment-resolved cytokine profiling in human fulminant viral myocarditis, ideally paired with viral genome status, has to our knowledge never been published, and until it is, any subtype scheme in this disease remains borrowed.

### 3.4 Underestimated regulators of innate immunity: natural killer cells and dendritic cells

Beyond the focus on macrophages and neutrophils, the role of natural killer (NK) cells is often overlooked. As early as the 1990s, immunoelectron microscopy demonstrated that perforin molecules could be directly released onto the surface of cardiomyocyte membranes, forming ring-shaped pores and causing injury (91). However, the net effect of NK cells is actually protective—they both limit viral replication to attenuate CVB3 and cytomegalovirus myocarditis, and restrain cardiac inflammation and fibrosis through regulation of eosinophil-associated chemokines (CCL11, CCL24, CXCL9, CXCL10) (92). When myeloid-derived suppressor cells impair NK cell function, viral load and myocardial injury are exacerbated, and the risk of chronicity increases (93). Perforin also exerts an intrinsic regulatory role in NK cell homeostasis (94). Dendritic cells (DCs) and granulocyte-macrophage colony-stimulating factor (GM-CSF) constitute another pathogenic axis. GM-CSF mediates autoimmunity by enhancing IL-6-dependent Th17 cell development (95) and promotes the formation of inflammatory dendritic cells (96); in the myocardium, it is precisely the type 2 classical dendritic cells (cDC2) loaded with α-myosin that trigger autoimmune responses (97). As a hub cytokine of the inflammatory network, GM-CSF forms a positive feedback loop with IL-1 and TNF, sustaining chronic inflammation (98).

## 4 Adaptive immunity: dysregulation and exhaustion – immunopathology in the acute phase (approximately days 3–14)

### 4.1 The infiltration paradox of cytotoxic T cells: viral clearance versus direct myocardial injury

Established evidence in viral myocarditis. Virus-specific CD8+ T cells are required for clearance of cardiotropic virus and are at the same time capable of killing cardiomyocytes; this is the infiltration paradox of adaptive immunity, and as a phenomenon it is well established. In CVB3 and cytomegalovirus infection the NK and T-cell compartments restrain viral replication, and their impairment by myeloid-derived suppressor cells raises viral load, worsens myocardial injury and increases the risk of progression to chronic disease (92, 93). Perforin-mediated pore formation on the cardiomyocyte membrane was demonstrated directly in myocarditis by immunoelectron microscopy (91), and perforin additionally governs NK-cell homeostasis itself (94). What has not been established in viral myocarditis is the clonal architecture of that response: no study has sequenced paired myocardial and circulating T-cell repertoires in human FVM, so the size, specificity and persistence of the responding clones remain unmeasured.

Cross-disease evidence and mechanistic extrapolation. The clonal detail that viral myocarditis lacks comes from checkpoint-inhibitor myocarditis, and it is applied here under the temporal restriction set out in Section 1.1: that condition models the antigen-driven, virus-negative end of the trajectory, not its opening days. Single-cell T-cell receptor sequencing has revealed that in immune checkpoint inhibitor-associated myocarditis, CD8^+^ T cells infiltrating the myocardium undergo significant clonal expansion, displaying a highly activated, cytotoxic phenotype with high expression of granzyme B and perforin (99). These clonally expanded effector CD8^+^ T cells show substantial overlap between the myocardium and peripheral blood, and their released granzyme B can directly cleave myocardial structural proteins and induce cardiomyocyte apoptosis (100).

Further mechanistic studies suggest that α-myosin may be a key autoantigen driving this autoreactive T-cell response: in Pdcd1^-^/^-^Ctla4^+^/^-^ mice, α-myosin-specific clonal effector CD8^+^ T cells predominate, and depletion of CD8^+^ (but not CD4^+^) cells improves survival (100). In human patients with fulminant immune checkpoint inhibitor-associated myocarditis, expandable α-myosin-specific T cells can also be detected (101). These cytotoxic T cells, bearing senescent/terminally differentiated phenotypes (e.g., CD57, KLRG1), may exert “bystander killing” effects beyond the requirement for viral clearance (102, 103). Classical autoimmune myocarditis models have also confirmed that CD4^+^ T-cell responses directed against cardiac myosin are sufficient to elicit significant myocardial inflammation in the absence of any infectious trigger (104). Synthesis, with two limits stated. All of the clonal data above come from settings in which no virus is replicating in the myocardium, so the trade-off that constitutes the paradox in FVM - killing that is simultaneously necessary for clearance - is absent from the models by design. And alpha-myosin-directed autoreactivity of this kind is a plausible mechanism for the second and third weeks and for progression to chronic inflammatory cardiomyopathy; it is not a plausible mechanism for hyperacute collapse, which in FVM occurs well before a clonal T-cell response could have expanded to that extent. The temporal placement of this evidence in **Figure 1B** follows from that argument.

### 4.2 Immune checkpoint “regulatory failure” and Th17/Treg imbalance

The PD-1/PD-L1 axis serves as a critical “brake” constraining T-cell overactivation. Pathological and clinical data from immune checkpoint inhibitor-associated myocarditis indicate that when this axis is disrupted or abnormally underexpressed in the myocardial microenvironment, T cells cannot be effectively suppressed, thereby triggering “bystander killing” and amplifying tissue injury; although the incidence of this complication is approximately 1%, its case-fatality rate can reach 25% to 50% (105, 106). Combined checkpoint blockade can even induce fulminant myocarditis (107). This entity illustrates unrestrained T-cell killing in a virus-free myocardium, and the PD-1/PD-L1 logic drawn from it is applied in this review to the adaptive phase of FVM rather than to the innate-dominant hyperacute window.

Evidence from autoimmune myocarditis, which maps onto the later part of the viral trajectory rather than onto its first days, indicates that the imbalance between helper T cell 17 (Th17) and regulatory T cell (Treg) populations is an important mechanism driving chronic inflammation and the transition to dilated cardiomyopathy. IL-6 drives Th17 differentiation via STAT3 and RORγt, and is essential for the initiation of experimental autoimmune myocarditis (108). In human myocarditis/dilated cardiomyopathy, cardiac myosin can act as an endogenous TLR ligand to stimulate CD14^+^ monocytes to produce pro-Th17 cytokines, and elevated Th17 cells are associated with heart failure (109). The NLRP3 inflammasome, myeloid-derived suppressor cells, and tissue microenvironment factors (such as tenascin-C) all participate in modulating the Th17/Treg balance, thereby influencing the persistence or resolution of inflammation (110–112). Because these findings derive from virus-free models and from chronic human disease, we read them as describing what happens to a subset of patients after viral clearance, and not as a mechanism operating at presentation.

### 4.3 A candidate immunosuppressive trajectory: what is established, what is borrowed

Established evidence in viral myocarditis. The direct evidence base here is thin, and we state its extent before building on it. Myeloid-derived suppressor cells expand in myocarditis in proportion to the severity of myocardial inflammation, restrain excessive T-cell responses, and under some conditions sustain low-grade inflammation instead of resolving it (111, 113); when they impair NK-cell function, viral load and myocardial injury both increase and the risk of chronicity rises (93). That an immunosuppressive counter-regulation exists in viral myocarditis is therefore established. What is not established is any of the clinical content usually attached to the term immunoparalysis: no published cohort has measured monocyte HLA-DR, serum IL-10 or rates of secondary infection serially in fulminant viral myocarditis, and consequently the incidence of the state, the features that predict it and its independent prognostic weight are all unknown in this disease.

A critical turning point during the acute phase is the transition of a subset of patients from a “cytokine storm” into what has been termed immunoparalysis — in FVM a candidate trajectory rather than an established disease phase. Sustained high-load antigen exposure and inflammatory cytokines induce massive expansion of myeloid-derived suppressor cells (MDSCs) and regulatory B cells (113). Clinically, these patients exhibit paradoxical elevations of serum interleukin-10 (IL-10) and transforming growth factor-β (TGF-β), along with downregulation of monocyte human leukocyte antigen-DR (mHLA-DR) expression, frequently falling below the <30% level used in critical-care studies. In sepsis cohorts this pattern has been interpreted as a shift from a hyperinflammatory state toward secondary infection and multi-organ dysfunction (114, 115); whether the same interpretation holds in FVM has not been tested.

It must be emphasized that the mHLA-DR <30% threshold, together with its associated incidence, risk factors, and prognostic weight, has been established almost entirely in sepsis and critical-illness cohorts (114–117); FVM-specific data on the incidence of immunoparalysis, the clinical or laboratory features that predict its onset, and its independent prognostic value are currently lacking. Extrapolation from sepsis is used here because the downstream molecular logic (IL-10–JAK–STAT3-driven monocyte deactivation) is shared, but this remains an assumption rather than a validated fact in FVM, and dedicated prospective studies measuring mHLA-DR trajectories in FVM are needed before this threshold can be used to guide therapy (see Section 5.3). Accordingly, wherever mHLA-DR appears in this review, including in the Abstract, **Figures 1**, **2**, Section 5.3 and Section 6.3, it should be read as an exploratory, association-level marker: it is not presented as predicting secondary infection in FVM, and it is not presented as a variable that can currently be used to start, continue or withdraw immunosuppression at the bedside.

**Figure 2:**
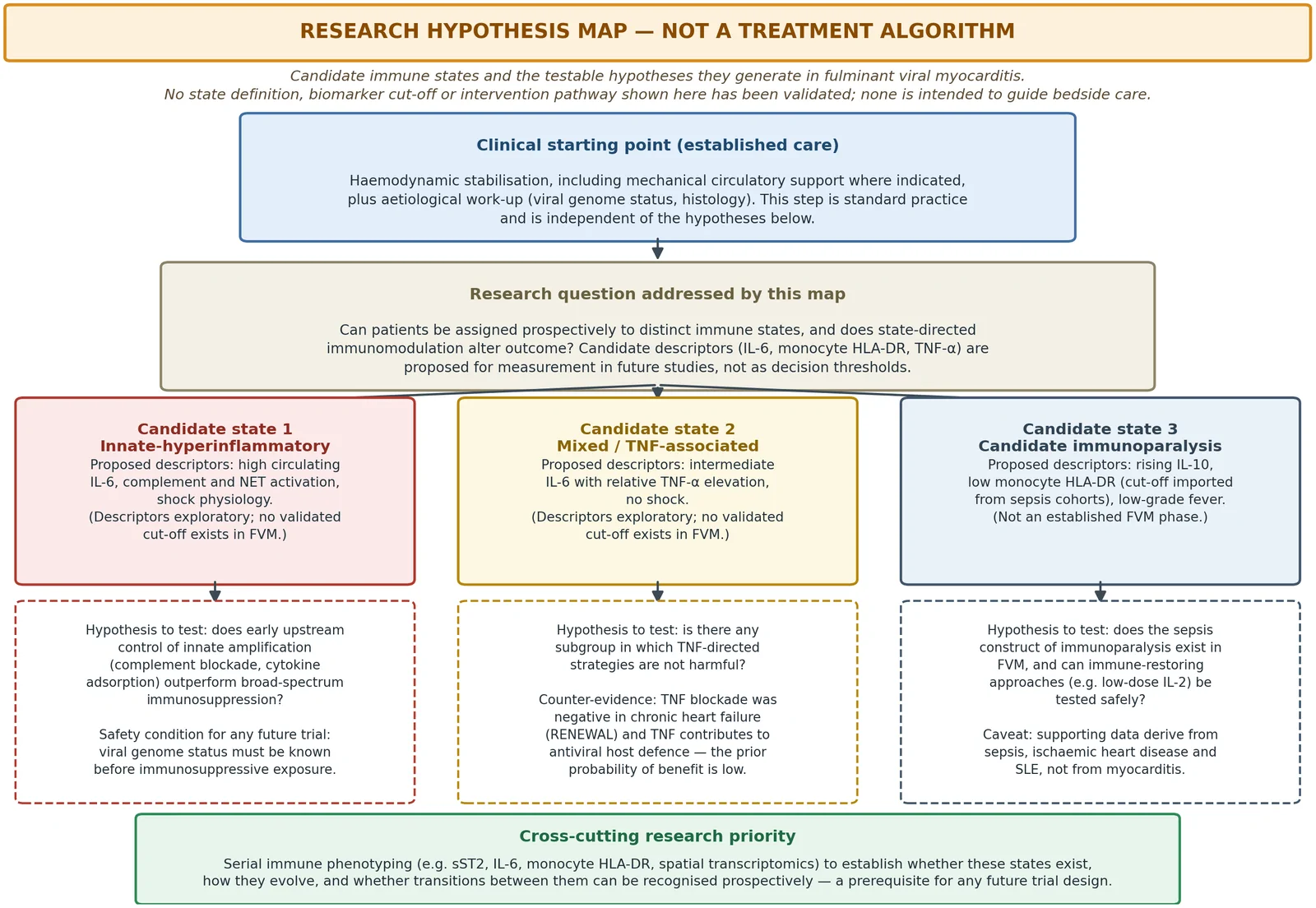
Research hypothesis map of candidate immune states in fulminant viral myocarditis. It is a hypothesis map, not a treatment decision tree, and specifies no actionable thresholds, drugs or sequences. Three candidate immune states are shown — innate-hyperinflammatory, mixed/TNF-associated, and candidate immunoparalysis — each paired with the research question it generates and with the principal objection to premature clinical use. Haemodynamic stabilisation and aetiological work-up, including determination of viral genome status, are shown as established care that is independent of the hypotheses below them; the safety requirement that viral status be known before any immunosuppressive exposure is stated within the figure. The biomarkers named (IL-6, TNF-alpha, monocyte HLA-DR, sST2) are proposed as descriptors for prospective measurement, not as decision thresholds: the IL-6 and HLA-DR values discussed in the text are exploratory and, in the case of HLA-DR, imported from sepsis cohorts. TNF-directed strategies are shown together with the negative RENEWAL result and the host-defence role of TNF, and low-dose IL-2 together with the fact that its supporting data come from ischaemic heart disease and systemic lupus erythematosus. Nothing in this figure is validated for bedside use (see Sections 6.3 and 7).

Cross-disease evidence and mechanistic extrapolation. The molecular logic of immunoparalysis is homologous to the “immunosuppressive phase” of sepsis: sustained IL-10 signaling, via the IL-10R–JAK–STAT3 pathway, inhibits antigen presentation and antimicrobial effector functions, induces CD14^+^HLA-DR^low^ immunosuppressive monocytes, and dampens T-cell activation (116, 117). Of note, MDSCs play a “plastic” role in myocarditis: their expansion correlates with the severity of myocardial inflammation, and they can both restrain excessive T-cell responses and, under certain conditions, sustain low-grade inflammation (111). Therefore, dynamic monitoring of mHLA-DR holds significant clinical value for identifying “when to stop immunosuppression and when to awaken immunity” (115).

Synthesis. Taking the two layers together, the most that can be claimed is a candidate trajectory: a subset of patients who survive the acute phase plausibly enter a counter-regulated state whose molecular machinery is documented in viral myocarditis but whose clinical expression has only been characterised in sepsis. In this review it is therefore treated as a candidate trajectory and not as a third stage of the disease. It is included nonetheless because decisions to continue or withdraw immunosuppression in fulminant myocarditis are being made now, in the absence of any marker of the patient immune state, and naming the gap generates the single most testable hypothesis in this review - that serial monocyte HLA-DR and IL-10 measurement in an FVM cohort will identify a subgroup in whom continued suppression is harmful. Until such a cohort exists, nothing in this subsection should be used at the bedside.

### 4.4 Humoral autoimmunity: the dual role of anti-heart antibodies as pathogenic and biomarker

Beyond cellular immunity, humoral autoimmunity serves as a bridge in the progression from myocarditis to dilated cardiomyopathy (DCM). Anti-heart autoantibodies, in patients who are virus-negative but autoantibody-positive, suggest potential benefit from immunosuppression, and non-antigen-specific immunoadsorption to remove these antibodies has been shown to improve ventricular function in some patients, indicating a functional role for these antibodies (118). Prospective family-based studies have demonstrated that cardiac-specific autoantibodies are detectable in approximately 60% of both familial and non-familial pedigrees, and can predict the development of DCM years before clinical and echocardiographic evidence appears (119). Antibodies targeting myosin and other cardiac antigens are repeatedly observed in myocarditis, DCM, and even Chagas heart disease, although their precise pathogenic weight remains incompletely elucidated (120). Based on this evidence, immunoadsorption therapy has been attempted to remove IgG, particularly anti-β1-adrenergic receptor antibodies, aiming to improve cardiac function and clinical status in heart failure patients (121). This line of evidence resonates with the immunosuppressive strategies for virus-negative subgroups, which will be discussed in Chapter 5.

### 4.5 Modulation of immune responses by host background: sex and hormones

The intensity and quality of immune responses are determined not solely by the pathogen and the myocardium; host background also shapes the trajectory of the inflammatory network, with sex being a long-underestimated effect modifier. Males not only have higher incidence but also more severe inflammation and a greater risk of progressing to DCM and myocardial fibrosis. Mechanistically, testosterone drives Th1-type responses via the androgen receptor and upregulates TLR4 expression on macrophages and mast cells, whereas estrogen, through the estrogen receptor, tends to promote anti-inflammatory Th2 polarization (122). In experimental autoimmune myocarditis, male animals predominantly exhibit a proinflammatory phenotype and collagen deposition, while females display more anti-inflammatory macrophages (123). Transcriptomic studies have attributed part of this difference to more robust mitochondrial homeostasis and ERRα-mediated metabolic advantages in female myocardium (124). The clinical implication is straightforward: two patients at the same phase and with the same cytokine thresholds, but of different sexes, may not share identical immune status. Therefore, interventions targeting the “inflammatory time window” must incorporate host sex and hormonal background into the interpretive framework, and the selective estrogen receptor modulators discussed in Chapter 5 represent an early attempt along this line.

## 5 Reconstructing precision intervention strategies based on the “inflammatory time window”

Before turning to phase-specific strategies, it is useful to make explicit which mechanisms reviewed in Sections 2–4 have been translated into candidate therapeutic targets below, and which have not. Identifiable, if variably supported, targeted interventions exist for complement C5a (vilobelimab, Section 5.1), the cytokine burden itself (hemadsorption, Section 5.1), the IL-6/gp130/JAK-STAT axis (tocilizumab, Section 5.2), the NLRP3-IL-1 axis (anakinra, Sections 5.2 and 5.4), TGF-beta-driven fibrosis (FAP-CAR-T, Section 5.5), and the Treg/IL-2 axis underlying immunoparalysis (low-dose IL-2, Section 5.3). TNF-alpha is discussed with particular caution throughout Section 5 because of its dual, context-dependent role in antiviral defense and myocardial homeostasis (135); this caution is foreshadowed here and is not specific to Section 5.2 alone. By contrast, several mechanisms discussed above have not been translated into specific interventions in this review and are flagged explicitly as non-targets: the Th17/Treg imbalance described in Section 4.2 is discussed as a pathogenic mechanism and a marker of disease activity rather than as a validated drug target in FVM, given the absence of FVM-specific trials of Th17-directed agents; anti-heart autoantibodies (Section 4.4) are discussed mainly as biomarkers and as the rationale for immunoadsorption in selected virus-negative, autoantibody-positive patients rather than as a broadly applicable target; and the sex- and hormone-related differences described in Section 4.5 currently lack sex-stratified interventional evidence in FVM, so selective estrogen receptor modulators are presented in Section 5.4 only as a hypothesis-generating direction rather than an actionable recommendation. This mapping is summarized, together with the corresponding level of evidence for each agent, in **Table 1**. A note on staging is also needed here. The 0-72 h, 3-14 d and >14 d intervals used as section headings are narrative devices for organising an immunological argument, not observed natural-history boundaries: no study has shown that FVM transitions occur on this clock, and individual patients move between immune states at very different speeds. Throughout Section 5, therefore, the operative criterion is the immune state described in each subsection (innate-dominant, adaptive-dysregulated, or immunosuppressed), and the calendar intervals should be read as approximate descriptive anchors only.

### 5.1 Hyperacute phase (approximately the first 72 hours): “fire-extinguishing” rather than “immunosuppression”

In the hyperacute phase, the primary therapeutic goal should be “fire-extinguishing”—rapidly reducing the inflammatory burden driven by innate immunity, rather than prematurely initiating broad-spectrum immunosuppression. Complement anaphylatoxin C5a acts upstream in neutrophil and monocyte chemotaxis, reactive oxygen species release, tissue factor expression, and microthrombus formation (125). The selective anti-C5a monoclonal antibody vilobelimab, in the phase 3 PANAMO randomized controlled trial for severe COVID-19, demonstrated reduced 28-day and 60-day mortality without inhibiting membrane attack complex formation and with limited impact on infection defense (126). It should be noted that PANAMO enrolled patients with severe COVID-19 rather than FVM, and vilobelimab currently holds FDA Emergency Use Authorization for that indication only; the discussion here should therefore be read as a mechanistic reference for upstream complement blockade rather than a treatment recommendation for FVM, pending dedicated studies in myocarditis. Its pharmacokinetic/pharmacodynamic analyses have confirmed effective C5a suppression and low immunogenicity (127, 128). This “upstream, selective” complement blockade strategy provides a reference for early interruption of neutrophil chemotaxis during the fulminant phase.

Blood purification represents another modality for “fire-extinguishing” in the hyperacute phase. Cytokine adsorption devices, such as CytoSorb, can rapidly reduce the burden of medium-molecular-weight mediators like IL-6 prior to corticosteroid use (129). In patients with cardiogenic shock supported by extracorporeal membrane oxygenation (ECMO), incorporation of CytoSorb into the ECMO or continuous renal replacement therapy circuit has been reported, with observed reductions in IL-6, procalcitonin, and C-reactive protein alongside decreased vasopressor requirements (129); the previously initiated ECMOsorb trial was designed to test this combination prospectively, but to date only its design and rationale, not clinical outcome data, have been published, so the observations above derive from real-world observational data rather than completed randomized evidence (130); its use has also been reported in cases of biventricular failure complicated by giant cell myocarditis (131). However, it must be recognized that the available evidence largely derives from case series and retrospective studies; whether adsorption therapy improves hard endpoints remains to be addressed by prospective randomized trials, and the optimal timing, duration, and target population have yet to be defined (129).

### 5.2 Acute phase (approximately days 3–14): “selective braking” rather than broad-spectrum suppression

Upon entering the acute phase, the strategy should shift from “fire-extinguishing” to “selective braking”—targeting specific pathways according to inflammatory subtypes while avoiding indiscriminate immunosuppression. For the IL-6-dominant subtype, the IL-6 receptor antagonist tocilizumab warrants attention: in refractory immune checkpoint inhibitor-associated myocarditis and COVID-19-related cardiogenic shock, rapid improvement in myocardial function following tocilizumab administration has been reported (132, 133). These reports originate from immune-checkpoint-inhibitor-associated myocarditis and COVID-19-related cardiogenic shock, not FVM; as discussed in Section 4.2, checkpoint-inhibitor myocarditis is driven mechanistically by PD-1/PD-L1-blockade-mediated loss of T-cell restraint, a pathway distinct from the virus-driven T-cell responses that characterize FVM. This experience should therefore be regarded as a cross-disease reference supporting biological plausibility rather than direct evidence for FVM, and any extrapolation should be made cautiously and only after excluding active viral replication. In large randomized trials of severe COVID-19, IL-6 receptor antagonists also improved survival in critically ill patients (134). However, the timing of administration should be guided by the dynamic slope of inflammatory markers such as C-reactive protein, rather than being applied uniformly (88). For the TNF-α-dominant subtype, TNF-blocking strategies using fusion proteins such as etanercept require cautious evaluation of their salvage value in very early intervention, given that TNF has dual roles in host antiviral defense and myocardial homeostasis (135). This caution is reinforced by the randomized RENEWAL programme (pooling RENAISSANCE and RECOVER), in which etanercept produced no clinical benefit and possibly worse outcomes in chronic heart failure (203), so any use of etanercept in FVM should currently be regarded as an unproven salvage option restricted to carefully selected, virus-excluded, TNF-dominant cases rather than a validated strategy.

The role of glucocorticoids warrants reassessment. The landmark TIMIC randomized controlled trial demonstrated that in virus-negative chronic inflammatory cardiomyopathy, a 6-month course of prednisone combined with azathioprine led to improved left ventricular function in 88% of patients, and this benefit persisted over a 20-year follow-up (136, 137). However, this conclusion is predicated on “virus-negative” status; in patients with active viral replication or a tendency toward immunoparalysis, broad-spectrum immunosuppression may be more harmful than beneficial. Therefore, a more prudent stance is that high-dose methylprednisolone pulse therapy (e.g., 500 mg/day for 3 days) should be reserved only for those with hemodynamic instability and markedly elevated IL-6, and should be avoided in patients showing immunoparalysis propensity (low mHLA-DR) (10, 115). It bears emphasis that TIMIC enrolled patients with chronic, virus-negative inflammatory cardiomyopathy treated with a 6-month oral prednisone-azathioprine regimen, which differs substantially in population, drug regimen, and time course from the short-course, high-dose intravenous pulse methylprednisolone considered here for acute FVM; TIMIC therefore provides supportive rationale for immunosuppression in virus-negative inflammatory heart disease in general, but should not be cited as direct randomized evidence for pulse corticosteroid therapy in the acute fulminant setting (136, 137).

Current guideline positioning should also be stated plainly here. The 2025 ESC Guidelines consider corticosteroids principally in fulminant myocarditis of non-infectious or autoimmune etiology — for example giant-cell, eosinophilic, sarcoid, or immune-checkpoint-inhibitor-associated disease — and in virus-negative inflammatory cardiomyopathy, rather than endorsing biomarker-triggered immunotherapy in virus-driven fulminant myocarditis (204). Our proposal to reserve pulse methylprednisolone for virus-negative, hyperinflammatory, hemodynamically unstable patients is therefore compatible with, but not equivalent to, guideline recommendation: the guidelines derive the indication from confirmed etiology and phenotype, whereas the IL-6 and mHLA-DR thresholds invoked in this review are exploratory refinements that have not been adopted in any guideline and remain unvalidated in FVM. Where the two frameworks diverge, current guideline recommendations should take precedence in clinical practice.

The experience with interleukin-1 pathway blockade reminds us of the importance of “stratification.” IL-1β is a key downstream product of the NLRP3 inflammasome and is theoretically an attractive target (138). However, the ARAMIS randomized controlled trial (enrolling 120 patients diagnosed by cardiac magnetic resonance rather than biopsy, predominantly low-risk and young male) showed that although short-course anakinra was safe, it failed to increase the number of days free from myocarditis-related complications (139, 140). This “negative” result does not negate the value of IL-1 blockade; rather, it underscores that broad anti-inflammatory approaches are unlikely to benefit unselected, low-risk myocarditis patients without identified viral status, and that potential gains are more likely concentrated in fulminant/hyperinflammatory, high-risk subgroups (140).

### 5.3 Subacute/reparative phase (approximately beyond day 14): “awakening” immunity and reversing immunoparalysis

In the reparative phase, if a patient shows features consistent with the candidate immunoparalysis state, one proposed direction is a shift of therapeutic emphasis from “suppression” to “awakening.” Low-dose interleukin-2 (IL-2) represents a paradigm of this approach: because resting Tregs constitutively express high-affinity IL-2 receptors at high levels, low-dose IL-2 at a thousandth of the oncological dose can selectively activate and expand Tregs without substantially activating effector T cells (141). We use the term “awakening” throughout this review as informal shorthand for this specific, Treg-mediated mechanism — restoration of regulatory capacity and, indirectly, of antigen-presentation competence — rather than to imply generalized stimulation of the immune system; low-dose IL-2 predominantly expands Tregs and has not been shown to directly reverse monocyte HLA-DR downregulation or global immune “paralysis” in FVM, and this distinction should guide interpretation of the term wherever it appears in this review, including in the Abstract, **Figures 1**, **2**, and Section 7. In autoimmune diseases such as systemic lupus erythematosus, low-dose IL-2 has been shown to expand Tregs and improve disease activity (142). In the cardiovascular field, randomized studies in ischemic heart disease (the LILACS/IVORY series) have confirmed that low-dose IL-2 can increase Treg numbers in patients (143); following acute coronary syndrome, low-dose IL-2 can also clonally expand effector Tregs with immunosuppressive and metabolic advantages (144). By extension, restoring Treg function is expected to suppress autoreactive B-cell production of anti-heart autoantibodies and attenuate chronic myocardial inflammation—however, caution is needed regarding the risk of capillary leak and arrhythmias at higher IL-2 doses, making dose selection critical (143). No FVM-specific trial has yet defined the mHLA-DR level, IL-10 concentration, or clinical timing at which low-dose IL-2 should be initiated; the regimens referenced above derive from ischemic heart disease, acute coronary syndrome, and systemic lupus erythematosus, and the consideration of low-dose IL-2 in FVM-associated immunoparalysis should therefore be regarded as hypothesis-generating rather than a validated, dose- and timing-specific clinical protocol pending dedicated studies.

Cell- and exosome-based therapies offer another frontier for the reparative phase. Exosomes derived from mesenchymal stem cells (MSCs) can deliver miR-146a-5p to target IRAK1 and inhibit NF-κB p65 nuclear translocation, thereby reducing cardiomyocyte apoptosis and inflammation (145, 146); in a sepsis-induced myocardial injury model, MSC-derived exosomes enriched with miR-146a-5p have also shown cardioprotective effects (147). These preclinical findings suggest that “precision delivery” using exosomes as vehicles and targeting key inflammatory pathways may serve as a complementary approach to reverse immunoparalysis and promote myocardial repair (148).

It must be emphasized that all the above pharmacological interventions are built upon the foundation of “maintaining perfusion via mechanical circulatory support.” Veno-arterial ECMO is the core modality for “bridging to recovery” in fulminant myocarditis (149). Data from the Chinese CSECLS registry show that the 90-day survival rate of adult fulminant myocarditis patients receiving ECMO support is approximately 72%, with pre-ECMO cardiac arrest, post-ECMO lactate, and pH being independent prognostic factors (150). Multicenter studies and systematic reviews further indicate that early initiation of mechanical circulatory support, timely biopsy for definitive diagnosis, left ventricular unloading, and anticoagulation management are key to improving outcomes (151, 152).

### 5.4 Targeted therapy for special histological subtypes and salvage strategies for refractory cases

Treating fulminant myocarditis as a single entity would miss the therapeutic windows offered by histological subtyping. Interleukin-1 is a key mediator of myocardial inflammation: in a case of fulminant myocarditis secondary to T-cell lymphoma, anakinra (IL-1 receptor antagonist) produced significant improvement within 24 hours of administration, with successful weaning from mechanical circulatory and respiratory support within 72 hours (153). In pediatric cohorts, subcutaneous anakinra was likewise associated with rapid recovery of cardiac function (154); refractory cases complicated by macrophage activation syndrome have also been salvaged with high-dose intravenous anakinra (155). Giant cell myocarditis, in contrast, is driven by T lymphocytes and carries a 1-year mortality or transplantation rate of approximately 70%; combined immunosuppression has been shown to significantly prolong transplantation-free survival (156). Registry-based data indicate that cyclosporine-based combination regimens have improved outcomes (157), and a JACC review has proposed a diagnostic and treatment algorithm (158), while mechanical circulatory support affords better survival benefit when immunosuppression is initiated first (159). Management of eosinophilic myocarditis depends on the underlying etiology; glucocorticoids are first-line, and for refractory or steroid-dependent cases, mepolizumab or benralizumab targeting IL-5 or its receptor can be used (160, 161); vigilance should be maintained even in the absence of peripheral eosinophilia, and thromboembolic prophylaxis should be considered (162).

For refractory, particularly immune checkpoint inhibitor-associated fulminant cases, stepwise escalation of immunosuppression is a practical choice. Tocilizumab has been used in cases with concomitant myositis/myasthenia-like manifestations (163); when patients respond poorly to high-dose corticosteroids, intravenous immunoglobulin, and plasma exchange, rituximab-mediated B-cell depletion has enabled weaning and recovery (164). Therapeutic plasma exchange can also rapidly remove circulating monoclonal antibody drugs themselves, reversing the condition (165). The evidence for intravenous immunoglobulin is inconsistent: retrospective studies suggest it may improve survival in fulminant (especially pediatric) cases (166, 167). At the mechanical support level, veno-arterial ECMO has become a mainstay for bridging fulminant myocarditis, with pediatric cohort discharge survival rates ranging from 53.8% to 83.3% (168); comparative studies in adults and children have identified pre-ECMO cardiac arrest as a common adverse prognostic factor (169), while single-center decade-long data also underscore the value of early identification of risk factors (170). Additionally, selective estrogen receptor modulators such as bazedoxifene have been shown to attenuate myocarditis in experimental models by modulating Th17 responses, providing clues for sex-directed interventions (171).

### 5.5 Engineered cell therapies for fibrosis: a crossover from oncology to cardiology

One of the core challenges in the reparative phase is fibrosis. Transforming growth factor-β (TGF-β) drives cardiac fibroblast-to-myofibroblast transdifferentiation and extracellular matrix deposition (172), while crosstalk between macrophages and fibroblasts further amplifies TGF-β/Smad signaling via IL-6 (173). Conventional anti-fibrotic approaches have proven insufficient, but engineered cell therapies have opened new avenues. Chimeric antigen receptor (CAR) T cells targeting fibroblast activation protein (FAP) can specifically eliminate pathological cardiac fibroblasts in mice, reducing fibrosis and restoring cardiac function (174). Taking a step further, delivery of modified mRNA via CD5-targeted lipid nanoparticles enables transient *in vivo* generation of anti-fibrotic CAR-T cells, preserving efficacy while avoiding persistent cytotoxicity (175). Such strategies are not in conflict with the “awakening immunity” concept advocated in this review for the reparative phase—the former eliminates pro-fibrotic effector fibroblasts, while the latter restores anti-infective immunity, and the two can be complementary in timing.

## 6 Multi-omics-guided “dynamic immune monitoring” and individualized decision-making

### 6.1 The cornerstone of histological diagnosis and its limitations

Any “dynamic immune monitoring” must be grounded in reliable etiological and histological characterization. Endomyocardial biopsy (EMB) has long been regarded as the histological gold standard, and the joint scientific statement from the American Heart Association, the American College of Cardiology, and the European Society of Cardiology provides biopsy indications across 14 clinical scenarios, among which fulminant course and non-response to empirical therapy are listed as reasonable indications (176). However, the limitations of the Dallas criteria have long been recognized: sampling error, inter-observer variability, and terminological confusion collectively reduce sensitivity (177). To improve consistency, the transatlantic pathology community has proposed the “Seaport” consensus criteria, which center on CD3 immunohistochemistry and grade according to the extent of infiltration and the degree of myocardial injury (178). Complementing histology, nasopharyngeal swab pathogen testing and peripheral blood molecular markers can aid in etiological subtyping and, to some extent, reduce reliance on invasive sampling (179). Within the framework of this review, the value of biopsy extends beyond definitive diagnosis—the presence or absence of viral genomes in myocardial tissue directly demarcates the safety boundary for immunosuppression, a point of decisive importance in the staging strategy discussed in Chapter 5.

This position is consistent with the 2025 ESC Guidelines for the management of myocarditis and pericarditis, which retain endomyocardial biopsy as the diagnostic reference standard and assign it a strong indication precisely in the clinical scenarios that define FVM — fulminant presentation, cardiogenic shock, life-threatening arrhythmia, or ventricular dysfunction refractory to standard therapy — because the histological and virological result determines whether immunosuppression can be given safely (204). The guidelines likewise place viral genome detection in myocardial tissue, rather than peripheral serology, at the centre of etiological classification, reinforcing the “virus-positive versus virus-negative” boundary that governs every therapeutic decision discussed in Section 5. Notably, the ESC framework organizes management around clinical phenotype and confirmed etiology rather than around circulating biomarker thresholds, and this difference in logic should be kept in view when interpreting the monitoring panel proposed in Section 6.3 and the conceptual model shown in **Figure 2**.

### 6.2 Imaging biomarkers: CMR parametric imaging and prognostic stratification

Cardiac magnetic resonance (CMR) has evolved from purely qualitative diagnosis to parametric quantification. The 2018 revised Lake Louise criteria incorporated T1 and T2 mapping and extracellular volume (ECV), requiring at least one T1-based and one T2-based criterion to be positive, and significantly improved sensitivity over the 2009 version (180). Validation cohorts have confirmed its superior diagnostic performance over the original criteria (181), and meta-analyses have further shown that native T1 mapping offers the highest diagnostic accuracy (182). Multiparametric studies using EMB as the reference standard, as well as applications in children and adolescents, support the robustness of the 2018 criteria (183, 184). Beyond diagnosis, late gadolinium enhancement (LGE) carries key prognostic information. The ITAMY multicenter study demonstrated that even in patients with preserved left ventricular ejection fraction, mid-wall LGE in the anterior septum portends worse outcomes (185); in a two-year follow-up of the fulminant phenotype, LGE patterns also held prognostic significance (186); in long-term follow-up of biopsy-proven viral myocarditis, LGE was the best independent predictor of all-cause and cardiovascular mortality (187); among those with infarct-like presentation, the transmural extent of LGE correlated with adverse events (188). Of course, not all studies have assigned independent prognostic value to LGE—in hemodynamically stable routine patients, only the initial ejection fraction decline was an independent predictor (189), underscoring that imaging markers must be interpreted in conjunction with clinical context.

Circulating nucleic acid markers offer a new tool for non-invasive differentiation. A study published in a top-tier journal identified a Th17-derived small non-coding RNA (human hsa-miR-Chr8:96) that distinguished acute myocarditis from myocardial infarction with an area under the curve of 0.927, and retained diagnostic value after adjustment for age, sex, ejection fraction, and troponin (190). Such molecular markers, when integrated with the combination of sST2, IL-6, and mHLA-DR discussed earlier in this chapter, are expected to translate “dynamic immune monitoring” from concept to actionable bedside phenotyping.

### 6.3 A “combined panel” of biomarkers

A single biomarker is insufficient to characterize the rapidly evolving immune status in FM. A feasible approach is to construct a three-dimensional monitoring system covering “fibrosis–inflammation–immunosuppression”: soluble suppression of tumorigenicity 2 (sST2) reflects fibrosis and myocardial stress, IL-6 reflects inflammatory burden, and mHLA-DR reflects the propensity toward immunosuppression/immunoparalysis (115, 135). The value of this combination lies in its “directional” information: elevated IL-6 signals a need for “braking,” whereas low mHLA-DR has been proposed, chiefly on the basis of sepsis and critical-care data, as a reason for caution about continued suppression; these are directional, association-level signals only, and the dynamic behaviour of the markers is offered as a research framework for identifying transitions rather than as a validated trigger for switching therapy (114).

None of the three markers in this triad currently provides a validated, treatment-guiding threshold in FVM. The IL-6 value of >100 pg/mL referred to in Sections 5.1-5.2 and in **Figure 2** is an exploratory cut-off adapted from cytokine-release-syndrome and severe COVID-19 literature; it has never been prospectively tested as a trigger for immunomodulation in myocarditis of any type, no FVM cohort has examined whether outcomes differ above and below it, and it should therefore be understood as a working value proposed for future study rather than a clinically established decision point. No validated sST2 threshold for triggering a specific intervention has been established in FVM, and sST2 is used here only as a qualitative indicator of fibrotic/mechanical stress trend. Similarly, mHLA-DR <30% derives from sepsis and critical-illness cohorts; its use as a point at which to withdraw immunosuppression or to consider low-dose IL-2 is discussed in Sections 4.3 and 5.3 as an extrapolated hypothesis, not as validated practice. This monitoring panel should accordingly be regarded as a structured framework for hypothesis generation and individualized clinical judgement rather than a biomarker-triggered treatment algorithm.

### 6.4 Clinical translation of spatial transcriptomics

Spatial transcriptomics is transforming our understanding of the “spatial architecture” of myocardial inflammation. Spatial multi-omics studies of healthy and diseased hearts have defined cellular microenvironments such as the epicardial immune defense niche and the ventricular myocardial stress niche (191). Applying this technology to endomyocardial biopsy tissue enables molecular-level discrimination between “inflammatory-infiltrative” and “edematous-necrotic” phenotypes: a spatial transcriptomic study of human myocarditis biopsies found that molecular perturbations in myocarditis extend far beyond leukocyte infiltrates, with even non-immune cells (cardiomyocytes) expressing immune-response genes traditionally considered “exclusive” to professional antigen-presenting cells (192). In giant cell myocarditis, spatial transcriptomics further revealed an immune-metabolic stratification distributed along the epicardial-to-endocardial axis, providing molecular evidence for judging inflammatory progression and determining whether to escalate ECMO support (193, 194). Two of the datasets cited in this subsection (192, 193) are currently available only as preprints and have not completed peer review; they are cited as emerging, provisional evidence and the conclusions drawn from them should be treated accordingly.

### 6.5 Perspectives on novel cell therapies and immunomodulation

Looking forward, therapeutic exploration in immune checkpoint inhibitor-associated myocarditis provides a template for “individualized immunomodulation.” A strategy combining the CTLA-4 agonist abatacept with the JAK inhibitor ruxolitinib, with dose individualization targeting a circulating monocyte CD86 receptor occupancy of >80%, has achieved “low mortality” reversal in multiple refractory/fulminant cases (195, 196). This paradigm of “biomarker-guided dosing and multi-pathway combination” embodies the concept of “dynamic phenotyping and real-time switching” advocated in this review (197). The host-background principle illustrated by immune-mediated myocarditis of other causes, in which pre-existing immune dysregulation and hormonal state shape individual susceptibility (198–200), applies equally to the viral disease: precision immunomodulation will have to incorporate host genetic and immune background, not phase alone (**Figure 3**).

**Figure 3:**
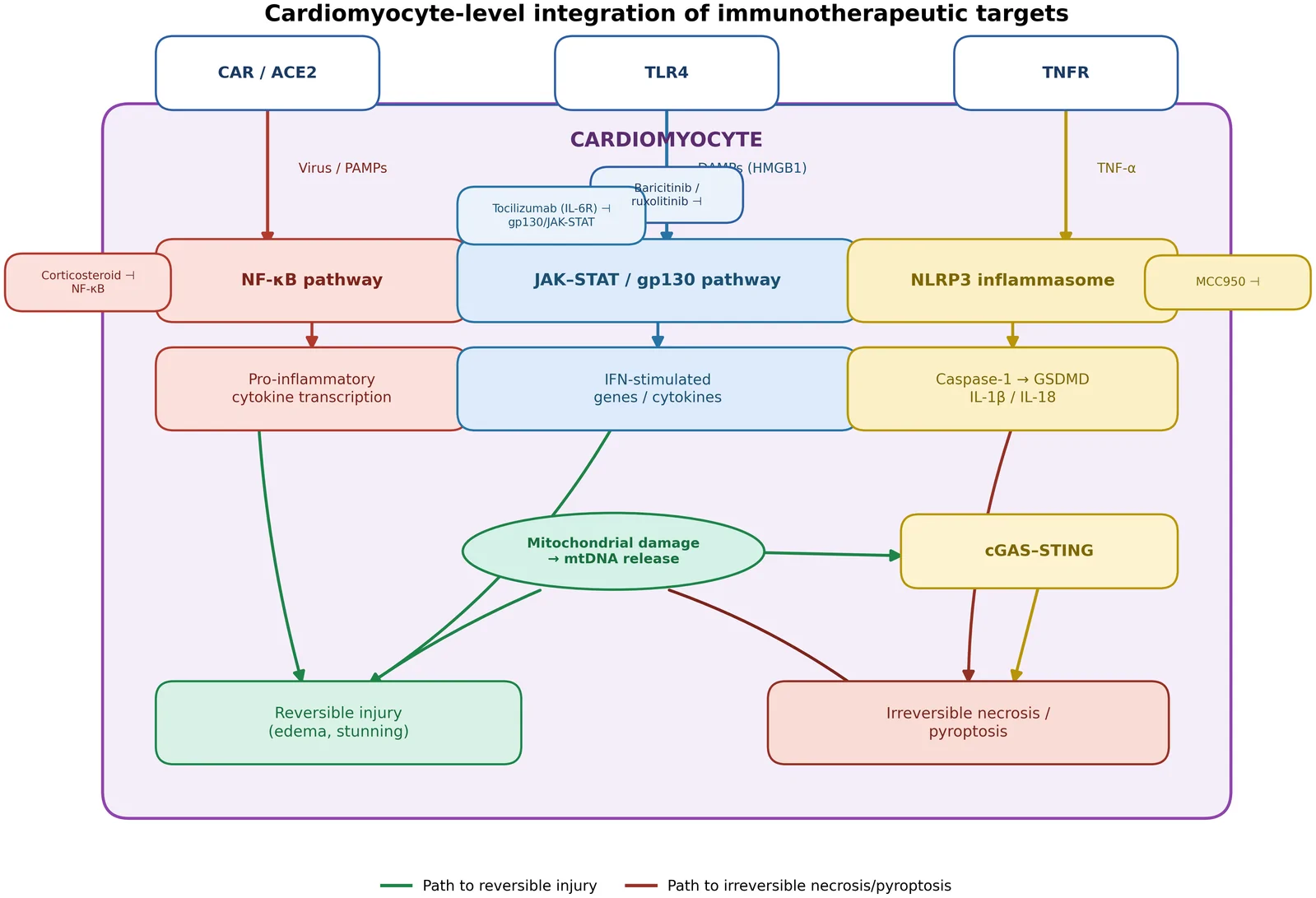
Integrated signaling map of immunotherapeutic targets at the cardiomyocyte level. Membrane receptors (CAR/ACE2, TLR4, TNFR) sense viral and damage signals. Three intersecting cytoplasmic pathways are shown: NF-kappaB (inhibited by corticosteroids), JAK-STAT/gp130 signaling (targeted by tocilizumab via IL-6 receptor blockade, and by baricitinib/ruxolitinib via direct JAK inhibition), and the NLRP3 inflammasome-pyroptosis axis (targeted by MCC950). Tocilizumab is shown acting on the gp130/JAK-STAT axis rather than on NF-kappaB, consistent with its mechanism of IL-6 receptor blockade. Mitochondrial damage releases mtDNA that activates cGAS-STING, amplifying interferon responses. Arrows distinguish reversible injury from irreversible necrosis/pyroptosis.

## 7 Conclusions and a proposed clinical management pathway

In summary, the core feature of fulminant viral myocarditis is the dramatic temporal evolution of the inflammatory network: from an innate immunity-dominated “cytokine storm” in the hyperacute phase, to the “dysregulation” of adaptive immunity in the acute phase, and, in a subset of patients, possibly toward a candidate immunoparalysis trajectory whose existence in FVM remains to be demonstrated. The claim the evidence supports is temporal; the spatial dimension and the immunoparalysis trajectory remain hypotheses awaiting direct data in FVM. Its prognosis does not depend on any single “magic bullet,” but rather on the clinician’s capacity for “dynamic phenotyping and real-time switching” of immune status (7, 10).

Accordingly, the authors propose — as a research scaffold for trial design rather than as clinical guidance — a stepwise framework organised around the “state–subtype” axis: on the premise of maintaining perfusion with mechanical circulatory support, the hyperacute phase focuses on “fire-extinguishing” through complement blockade and cytokine adsorption; the acute phase employs “selective braking” based on IL-6/TNF-dominant subtypes and the presence or absence of shock, with glucocorticoids strictly indicated for “virus-negative, hyperinflammatory, hemodynamically unstable” patients; and the reparative phase aims to “awaken” immunity and reverse immunoparalysis via low-dose IL-2, exosome-based therapies, and other approaches (15, 136, 143). Throughout the entire course, dynamic immune monitoring represented by the “sST2 + IL-6 + mHLA-DR” triad and spatial transcriptomics may be explored as a research tool (115, 192). The greatest gap in current evidence is that most immune-targeted strategies remain at the level of observational studies and case series, lacking high-quality randomized controlled trials specifically targeting fulminant/high-risk subgroups; the negative results of “all-comer” trials such as ARAMIS precisely underscore the necessity of “precise stratification” rather than “blanket anti-inflammation” (139, 201). Future research should focus on the core question of “how to deliver immunomodulation at the right time, to the right patient, and with the right intensity,” and the “inflammatory time window” framework proposed herein may serve as a conceptual starting point for designing such trials (2, 202).

This framework should also be read alongside current clinical guidance. The 2025 ESC Guidelines for the management of myocarditis and pericarditis, which introduce the umbrella term “inflammatory myocardial and pericardial syndromes,” similarly emphasize phenotype- and etiology-guided management, reserve immunosuppression and corticosteroids for fulminant non-infectious presentations, refractory ventricular dysfunction, or confirmed autoimmune etiology rather than for biomarker-triggered algorithms, and do not endorse the biomarker-defined thresholds proposed in **Figure 2** (204). Accordingly, **Figure 2** should be understood as a hypothesis-generating conceptual model synthesizing mechanistic and cross-disease evidence, not as a validated clinical decision algorithm; the fixed 0-3, 3-14, and >14-day phase boundaries used throughout this review likewise represent a conceptual scaffold for organizing the immunological narrative rather than clinically validated treatment windows, and both should be tested prospectively before any bedside application.

Evidence-source key: entries in the “Level of evidence” column derived directly from randomized trials or dedicated cohorts in myocarditis are indicated accordingly; all other entries represent evidence extrapolated from COVID-19, sepsis, ischemic/chronic heart disease, oncologic cytokine-release syndrome, or preclinical models, and are explicitly discussed as such, and as reference rather than recommendation, in the corresponding subsections of Section 5. Rows whose evidence derives from checkpoint-inhibitor-associated or autoimmune myocarditis (methylprednisolone, tocilizumab, abatacept plus ruxolitinib, rituximab and plasma exchange) should be read as informative for the acute-to-reparative interval of FVM, after viral replication has waned, rather than for the hyperacute window; rows derived from sepsis, ischaemic heart disease or oncologic cytokine-release syndrome carry no implied temporal mapping at all.
